# Serum Levels of Tumor Necrosis Factor-α and Interleukins in Children with Congenital Heart Disease

**Published:** 2017-01

**Authors:** Noor Mohammand Noori, Iraj Shahramian, Alireza Teimouri, Behrooz Keyvani, Maziar Mahjoubifard

**Affiliations:** 1 *Children and Adolescents’ Health Research Center, Zahedan University of Medical Sciences, Zahedan, Iran.*; 2 *Zabol University of Medical Sciences, Zabol, Iran.*

**Keywords:** *Tumor necrosis factor-alpha*, *Interleukins*, *Child*, *Heart defects, congenital*

## Abstract

**Background: **Levels of anti-inflammatory cytokines in blood have a positive relationship with congenital heart disease (CHD). We sought to assess the difference in serum cytokines levels between children with and without CHD.

** Methods: **We recruited 60 patients with CHD and 30 healthy children, from 2013 to 2014. Patients with primary pulmonary hypertension; metabolic diseases; renal, endocrine, and chronic inflammatory diseases; fever; infection in the preceding 3 weeks; and malnutrition were excluded. Participants’ demographic data were measured, and their cardiac diseases were diagnosed via echocardiography. Serum levels of tumor necrosis factor (TNF)-α, interleukin (IL)-6, and IL-18 were measured via ELISA.

**Results:** Mean age of the participants was 4.28 ± 3.44, 3.12 ± 3.87, and 3.30 ± 3.61 years in the cyanotic, acyanotic, and control groups, respectively (p value = 0.414). Mean values of TNF-α (p value < 0.001), IL-6 (p value < 0.001), IL-18 (p value = 0.030), right ventricular pressure (p value < 0.001), and pulmonary pressure (p value = 0.015) were higher in the case group, while the BMI was higher in the controls (p value < 0.001). Mean values of TNF-α (p value < 0.001), IL-6 (p value < 0.001), and right ventricular pressure (p value < 0.001) were significantly higher in the cyanotic children, whereas the BMI was higher in the controls (p value < 0.001). Levels of TNF-α and IL-6 had significant correlations with right ventricular pressure.

**Conclusion**: The present study showed a differed serum cytokines levels between children with and without CHD.

## Introduction

Congenital heart disease (CHD) occurs in 4 to 50 from 1000 live births.^1^ Cytokines have great immunologic actions and are considered important catabolic factors.^2^^, ^^3^ Many epidemic studies have shown that the levels of anti-inflammatory cytokines in blood have a positive significant relationship with CHD even in patients without a history of this disease.^4^ Varieties of cardiac malformations affect feeding and growth retardation in children with CHD.^5^ It seems that cytokines such as tumor necrosis factor alpha (TNF-α), interleukin (IL)- 6, IL-10, IL-12, and IL-18 are important mediators of the cachectic process in patients with CHD; this relationship, however, has yet to be fully confirmed.^6^ Patients with CHD have higher levels of serum TNF-α and IL- 6 than controls, but this is not clear in the case of IL-10 and IL-12. Also, the levels of TNF-α and IL-6 in patients with cyanosis are higher than those in acyanotic individuals.^7^ 

The cardiac cachexia syndrome in children with chronic congestive heart failure or chronic hypoxemia may oc-cur due to shunting. Among cytokines, TNF-α, IL-6, and IL-10 are secreted secondary to several chronic diseases. In addition, they play a major role in cardiac cachexia and exacerbate prognosis.^8^ Many studies have been con-ducted on the relationship between CHD and cytokine levels in cyanotic and acyanotic individuals.^9^ In patients with CHD, ghrelin, leptin, TNF-a, and interleukins are thought to have a regulating role in feeding, growth, weight, and energy.^10^ CHD also is a cause of malnutrition and growth retardation in children.^11^ As a biomarker, IL-18 is introduced for early diagnosis and prognosis in patients with acute kidney injury in the intensive care unit. Malnutri-tion is caused by several factors that decrease or increase the magnitude of the required energy in patients with CHD.^12^^, ^^13^ Moreover, clinical observations have demonstrated that the level of IL-18 in urine as a biomarker can be drawn upon for the early diagnosis of acute kidney injury after cardiac surgery.^14^^, ^^15^ IL-12 is an im-portant cytokine produced primarily by antigen-presenting cells and plays an important role as a regulator of the immune response of natural killer cells.^16^^, ^^17^ Since CHD exerts a considerable impact on the education and normal life of children and because it is treatable and curable, we sought to investigate its relationship with inflammatory cytokines. Therefore, the current study aimed to assess the differences in the excretion levels of cytokines between children with and without CHD.

## Methods

In this case-control study, 60 patients (30 cyanotic and 30 acyanotic; age range = 1 mo to 15 y) were compared with 30 age- and gender-matched healthy children. Patient selection was done using accessibility sampling with the Cochran formula in accordance with studies by Yilmaz et al.^18^ and Afify et al.^7^ Twenty-seven children were considered for each of the 3 groups considering the following parameters: Z_1-α/2_ = 1.96 and Z_1-β_ = 0.85, µ_1_ = 8, µ_2_ = 11 and S_1_ = 3.4, S_2_ = 4.1; where index 1 is related to the controls and 2 to the patients.  We added 3 persons in each group for more accuracy.

The present study was performed in Ali Asqar Clinic and the pediatric ward of Ali ebne Abi Talib Hospital, affiliated to Zahedan University of Medical Sciences, from 2013 to 2014. The study protocol was approved by the Research Ethics Committee of Zahedan University of Medical Sciences, and informed consent was obtained from the parents before enrollment in the study. Patients with primary pulmonary hypertension; metabolic diseases; renal, endocrine, and chronic inflammatory diseases; and malnutrition were excluded. Some common causes of increasing these mediators particularly in children are fever and infection in the recent 3 weeks. Therefore, this criterion was used as an exclusion factor. All the participants’ height, weight, age, sex, and body mass index (BMI) were recorded in a specific form. Weight in children < 2 years was measured using the Japanese MIKA scale factor with an error of 10 g and in children > 2 years by constructing the RASA scale factor with an error of 100 g. Height was measured in children < 2 years in the supine position on a scaled wooden table and in those > 2 years in the standing position with a balance using a scaled ruler. The BMI was calculated based on the following equation: weight (kg)/ height^2^ (m^2^). Cardiac diseases were diagnosed via echocardiography (My lab 60, Italy) with a 3 to 8 MHz transducer.

After obtaining consent from the parents, we carried out blood sampling. The patients’ samples were 3 cc and were taken in the cath lab via vessel catheters; they were kept for 60 minutes at room temperature. Separated serum was kept at -70 ˚C in a laboratory freezer. For the 30 subjects in the control group, statistical methods were used to generate right ventricular (RV) and pulmonary artery (PA) pressure randomly. We considered mean and standard deviation as 25 and 5 for RV pressure and 22 and 4 for PA pressure, respectively.^9^ After complete data collection, all the samples were transferred to the biochemistry laboratory in compliance with the cold chain. Finally, the levels of serum TNF-α, IL-6, IL-10, IL-12, and IL-18 were measured via ELISA using a kit by Bender MedSystems (U.S.).  Ethical codes of 3, 8, and 17 were observed. The statistical analyses were conducted using SPSS, version 20, and a p value = 0.05 was considered statistically significant. In the absence of a normal distribution, nonparametric tests and in the case of normality, parametric tests were employed. The Mann–Whitney *U*-test and the Kruskal-Wallis test-along with the independent *t*-test, ANOVA, and the Pearson correlation test-were used.

## Results

In the present study, 90 subjects were allocated to 3 groups (n = 30 in each group). There were 15 (50.0%), 17 (56.7%), and 16 (53.3%) females in the cyanotic, acyanotic, and control groups-respectively. Crosstab analysis using a contingency coefficient of 0.054 with a p value = 0.875 showed that the participants were matched in terms of gender.

The mean age was 4.28 ± 3.44 years in the cyanotic group, 3.12 ± 3.87 years in the acyanotic group, and 3.30 ± 3.61 years in the control group (F = 0.890; p value = 0.414), which revealed nonsignificant  differences in the mean of age between the 3 groups. This result showed that the 3 groups of participants were matched in terms of age.

The mean age of both case groups was 3.68 ± 3.71 years. The mean BMI in the cyanotic and acyanotic case groups was 15.17 ± 2.77 kg/m^2^ and 14.54 ± 2.66 kg/m^2^, respectively, and it was 14.86 ± 2.72 kg/m^2 ^and 18.86 ± 2.43 kg/m^2^ for the controls. In the cyanotic group, tetralogy of Fallot was observed in 20 patients, tricuspid atresia in 2, transposition of the great arteries in 2, total anomalous pulmonary venous connection in 1, complete atrioventricular septal defect in 5, and pulmonary hypertension in 6. In the acyanotic group, atrial septal defect was detected in 7 patients, ventricular septal defect in 17, pulmonary atresia with ventricular septal defect in 2, mitral valve prolapse in 1, pulmonary stenosis in 3, and pulmonary hypertension in 14. 

The one-sample Kolmogorov–Smirnov normality test revealed that the majority of the basic variables did not have a normal distribution. The results of the Kolmogorov–Smirnov test showed that TNF-α (p value < 0.001), IL-6 (p value = 0.001), IL-10 (p value < 0.001), IL-12 (p value < 0.001), IL-18 (p value < 0.001),  age (p value = 0.001), weight (p value = 0.027), height (p value = 0.035), RV pressure (p value = 0.007), and PA pressure (p value < 0.001)  did not have a normal distribution.

The nonparametric and independent Mann–Whitney tests were used to compare TNF-α, IL-6, IL-10, IL-12, IL-18, RV pressure, PA pressure, and the BMI between the case and control groups (Table 1). The mean value of TNF-α for the case and control groups was 56.6l pg/mL and 23.27 pg/mL, respectively. The TNF-α level for the case group was significantly higher than that for the control group. Similar trends could be observed for IL-6 pg/mL (55.28 mmHg vs. 25.95 mmHg), IL-18 (49.73 pg/mL vs. 37.05 pg/mL), RV pressure (60.05 mmHg vs. 16.40 mmHg), and PA pressure (50.20 mmHg vs. 36.10 mmHg), whereas the mean value of the BMI was significantly higher in the controls (34.08 kg/m^2^ vs. 68.35 kg/m^2^). The nonparametric Kruskal–Wallis test was applied to compare the values of TNF-α, IL-6, IL-10, IL-12, IL-18, RV pressure, PA pressure, and the BMI between the cyanotic, acyanotic, and control groups (Table 2). The mean value of TNF-α in the cyanotic, acyanotic, and control groups was 157.62, 43.76, and 24.09 pg/mL, respectively, and this difference was higher in the cyanotic group, followed by the acyanotic group (χ^2 ^= 57.82; p value < 0.001). The same pattern was observed for IL-6 with the corresponding values of 73.57, 36.98, and 25.95 pg/mL (χ^2^ = 54.70; p value < 0.001); for RV pressure with the respective values of 73.22, 46.88, and 16.40 mmHg; and for the BMI with the corresponding values of 36.37, 31.78, and 68.35 kg/m^2^. As regards the BMI, the highest value was observed among the controls, followed by the cyanotic patients.

Table 3 depicts the relationships between the parameters in the study for both cyanotic and acyanotic patients. There was a rise in TNF- α, IL-6 (Pearson correlation = 0.477; p value < 0.001), and RV pressure (Pearson correlation = 0.435; p value < 0.001); this association was statistically significant. IL-6 had a significant correlation with RV pressure (Pearson correlation = 0.567; p value < 0.000).  Moreover, the table shows a significant correlation between IL-10 and IL-12 and IL-18 (Pearson correlation = 0.261; p value = 0.044 and Pearson correlation = 303; p value = 0.018, respectively). RV pressure had no significant correlation with PA pressure, and nor was it correlated with the BMI. The table also demonstrates a significant correlation between weight and height in the study population. 

**Table 1:** Comparison of the mean values of the parameters between the case and control groups

Parameters	Median	Mean Rank	Interquartile Range	Sum of Ranks	Mann–Whitney U	P value
TNF-α (pg/mL)						< 0.001
Case	73.5	56.6	70.8	3397.0	233.0	
Control	20.0	23.3	12.8	698.0		
IL6 (pg/mL)						< 0.001
Case	8.0	55.3	8.6	3316.5	313.5	
Control	3.0	26.0	1.4	778.5		
IL10 (pg/mL)						0.053
Case	3.4	41.7	1.7	2504.0	674.0	
Control	4.0	53.0	1.6	1591.0		
IL12 (pg/mL)						0.068
Case	1.9	42.0	0.8	2517.0	687.0	
Control	2.3	52.6	1.2	1578.0		
IL18 (pg/mL)						0.030
Case	18.0	49.7	102.5	2983.5	646.5	
Control	15.0	37.1	45.3	1111.5		
Weight (kg)						0.212
Case	9.5	43.1	8.4	2584.5	754.5	
Control	11.5	50.4	8.3	1510.5		
Height (m)						0.482
Case	0.8	46.9	0.4	2812.0	818.0	
Control	0.8	42.8	0.5	1283.0		
Body mass index (kg/m^2^)						< 0.001
Case	14.3	34.1	2.6	2044.5	214.5	
Control	18.9	68.4	3.7	2050.5		
Right ventricular pressure (mmHg)						< 0.001
Case	70.0	60.1	57.0	3603.0	27.0	
Control	23.5	16.4	3.0	492.0		
Pulmonary artery pressure (mmHg)						0.015
Case	18.0	50.2	15.0	3012.0	618.0	
Control	15.0	36.1	4.0	1083.0		

**Table 2:** Comparison of the mean values of the parameters between the case (cyanotic and acyanotic) groups and the control group

Parameters	Median	Interquartile Range	Ranks	χ^2^	P value
TNF-a (pg/mL)				57.82	< 0.001
Cyanotic	98.0	58.5	73.6		
Acyanotic	31.5	31.4	39.7		
Control	20.0	12.8	23.3		
IL6 (pg/mL)				54.70	< 0.001
Cyanotic	12.5	4.6	73.6		
Acyanotic	3.7	3.2	36.9		
Control	3.0	1.4	25.9		
IL10 (pg/mL)				4.11	0.128
Cyanotic	3.4	1.2	39.7		
Acyanotic	3.4	2.0	43.7		
Control	4.0	1.6	53.0		
IL12 (pg/mL)				3.34	0.188
Cyanotic	1.9	0.7	41.9		
Acyanotic	2.0	0.8	41.9		
Control	2.4	1.2	52.6		
IL18 (pg/mL)				4.87	0.088
Cyanotic	183.0	109.3	51.1		
Acyanotic	176.0	107.0	48.4		
Control	151.0	45.3	37.1		
Weight (kg)				3.44	0.179
Cyanotic	10.3	9.1	47.7		
Acyanotic	9.0	5.2	38.5		
Control	11.5	8.3	50.4		
Height (m)				2.02	0.364
Cyanotic	0.8	0.4	51.0		
Acyanotic	0.8	0.3	42.7		
Control	0.8	0.4	42.8		
Body mass index (kg/m^2^)				34.90	< 0.001
Cyanotic	14.6	2.6	36.4		
Acyanotic	11.0	2.6	31.8		
Control	18.9	3.7	68.4		
Right ventricular pressure (mmHg)				71.35	< 0.001
Cyanotic	100.0	26.0	73.2		
Acyanotic	45.0	26.0	46.9		
Control	23.5	3.0	16.4		
Pulmonary artery pressure (mmHg)				5.92	0.052
Cyanotic	16.0	29.0	49.6		
Acyanotic	20.0	11.0	50.9		
Control	15.0	4.0	36.1		

**Table 3:** Correlation between the studied variables

Variables	Statistics	TNF	IL6	IL10	IL12	IL18	Weight	Height	Right Ventricular Pressure	Pulmonary Artery Pressure
IL6	PC	0.477								
	P value	0.000								
										
IL10	PC	-0.116	-0.177							
	P value	0.378	0.176							
										
IL12	PC	-0.045	0.147	0.277						
	P value	0.735	0.261	0.032						
										
IL18	PC	0.009	0.211	0.261	0.303					
	P value	0.947	0.105	0.044	0.018					
										
Weight	PC	0.135	0.033	-0.121	-0.044	-0.084				
	P value	0.304	0.805	0.359	0.738	0.521				
										
Height	PC	0.126	-0.005	-0.094	-0.026	-0.146	0.959			
	P value	0.338	0.967	0.476	0.842	0.265	0.000			
										
Right ventricular pressure	PC	0.336	0.567	-0.188	0.021	0.170	0.245	0.176		
	P value	0.009	0.000	0.151	0.872	0.194	0.060	0.177		
										
Pulmonary artery pressure	PC	-0.102	0.108	-0.045	0.054	-0.032	0.020	-0.011	0.234	
	P value	0.436	0.413	0.733	0.684	0.807	0.882	0.932	0.071	
										
Body mass index	PC	0.031	0.151	-0.097	-0.056	0.320	-0.200	-0.425	0.170	0.053
	P value	0.816	0.251	0.461	0.669	0.013	0.125	0.001	0.194	0.687

TNF, Tumor necrosis factor-alpha; IL, Interleukin; PC, Pearson correlation

**Table 4:** Analysis of variance (ANOVA) results for arterial blood gas (O_2_ saturation) in the groups*

Groups	Mean	Standard deviation
Cyanotic	59.47	15.14
Acyanotic	91.03	2.77
Control	95.70	1.47
Total	82.07	18.43

* F = 146.31, P < 0.001

The mean O_2_ saturation with arterial blood gas in the cyanotic patients was lower than that in the acyanotic and control groups (F = 146.31; p value < 0.001) (Table 4). In the multiple comparisons in the Tukey follow-up test in ANOVA, the mean O_2_ saturation was significantly different between the control and acyanotic groups and between the control and cyanotic groups (p value < 0.001).

## Discussion

A case-control study was performed on subjects, allocated to cyanotic, acyanotic, and controls. In all these groups, there was a higher number of females. The mean age was higher in the cyanotic patients. The mean BMI was much higher for the controls. The results demonstrated a rise in the serum levels of TNF-α, IL-6, and IL-18 as well as RV pressure and PA pressure in the cardiac patients compared to the control group, whereas there was a drop in the BMI. The correlations between RV pressure and TNF-α, IL-6, IL-18, and the BMI were statistically significant, while PA pressure had a significant correlation with IL-6 and RV pressure.

CHD is a common cause of malnutrition and growth disorders in children. Hallioglu et al.^19^ concluded that a rise in metabolism was a major factor in growth reduction due to CHD, particularly in cyanotic patients. We found that the BMI as a growth retardation indicator decreased significantly in the patients compared to the controls, with this metabolism variable being higher among the cyanotic patients. Yilmaz et al.^18^ emphasized that an insufficient caloric intake in CHD patients was the main cause of cachexia, while Arvat et al.^20^ showed that this problem was secondary to parental roles. Yilmaz et al.^18^ reported that the degree of growth retardation had no correlation with the severity of hypoxia and cardiac cachexia, which begot a decrease in muscle strength and weakened the immune function. This result is dissimilar to our findings insofar as the lowest level of the BMI was recorded among the acyanotic patients. The results of 2 studies by Cummings et al.^21^^, ^^22^ showed TNF-α as a cytokine with a strong and effective impact on cachexia in all kinds of illnesses; their results, however, did not confirm this relationship in cardiac patients. In our study, a rise in the BMI was followed by a decrease in TNF-α in the control group, which chimes in with the results of the studies by Cummings et al.^21^^, ^^22^ in this specific term. Dinleyici et al.^23^ reported that cardiac cachexia was allied to a lower BMI in children with CHD and that it caused changes in left ventricular ejection fraction. We observed a decrease in the BMI in the CHD patients, especially the acyanotic patients, by comparison with the control group, which provides evidence for the impact of circulating TNF-α on the inadequate intake of calories and protein as well as increased catabolism in the body.

Cytokines are secreted by the body’s cells in response to stress, hypoxia, and tissue destruction. They gradually release their effects on the body’s organs and cause cachexia, weakness, and malnutrition. Research shows that the ingestion of apoptotic cells is followed by the secretion of a large number of anti-inflammatory cytokines.^24^ Yilmaz et al.^19^ observed an increase in the serum levels of TNF-α in their patients compared to their healthy controls. The authors also reported that the rise in the serum levels among their cyanotic patients had no relationship with the BMI and that their patients had more weight and growth disorders. We found a significant increase in the serum levels of TNF-α among our cyanotic and acyanotic patients in comparison to our controls. Additionally, our results demonstrated that growth retardation was more pronounced in the acyanotic group. Stocker et al.^25^ measured the levels of TNF-α, IL-6, and IL-10 in patients after cardiopulmonary bypass at 24 ˚C and 34 ˚C and obtained similar results between their study groups. We found a rise in the levels of TNF-α and IL-6 among the CHD patients-especially in the cyanotic group-whereas there was no difference as regards the levels of IL-10. Aside from different methodologies drawn upon by our study and that by Stocker et al.,^25^ The results are also comparatively dissimilar. Lopes et al.^26^ reported that the plasma levels of IL-6 and IL-10 and pulmonary hypertension were significantly elevated in their patients with CHD compared to their controls. The authors reported that TNF-α exhibited no differences between the patients and controls. In contrast, our results demonstrated no significant differences in IL-6 and IL-10 but a significant difference in TNF-α between the case and control groups.  Lopes et al.^26^ also reported that there was no correlation between TNF-α and IL-10 and variables such as age, gender, and clinical presentation. Nevertheless, the results of the present study revealed that TNF-α and IL-18 were correlated with RV pressure in our CHD patients. According to Lopes et al.,^26^ IL-6 was correlated with IL-18, RV pressure, PA pressure, and the BMI-while IL-12 had no significant correlation with all the variables except IL-18. Cheung et al.^27^ measured and compared the levels of cytokines such as IL-6, IL-8, IL-10, and TNF-α at 3 and 6 hours after cardiac surgery in patients with CHD and observed no significant differences between their case and control groups. In contrast, we focused on cyanotic and acyanotic diseases and observed differences in this regard between our case and control groups. This discordance in the results must be in consequence of the application of different methodologies.

Cummings et al.^21^ found that an increase in TNF-α was due to heart disease. Their finding does not tally with our results inasmuch as some patients live with left-to-right shunts in severe degrees of heart failure. Afify et al.^7^ reported that the mean serum levels of TNF-α and IL-6 in their cyanotic and acyanotic patients were significantly higher than those in their controls and that the mean BMI did not show any difference. In our study, the mean serum levels of TNF-α, IL-6, and IL-18 were significantly higher in the case group than in the control group, which is similar to the results of the study by Afify et al.^7^ Nonetheless, the BMI was significantly lower in our study population (particularly in the acyanotic patients). Sharma et al.^28^ measured the levels of TNF-α, IL-6, and IL-10 in adult patients with CHD divided into 3 groups of asymptomatic patients, patients with mild symptoms, and patients with moderate/severe symptoms and observed no differences in IL-10 between the patients with CHD and the controls. The authors also reported that the levels of TNF-α and IL-6 were different between these groups and concluded that a rise in the severity of diseases was allied to a rise in the levels of TNF-α and IL-6 and a drop in the level of IL-10. These results are concordant with the results of the present study. Madhok et al.^29^ assessed TNF-α, IL-6, IL-8, IL-10, and IL-12 and reported that IL-6, IL-8, and IL-10 had a significant decrease on the 2nd and 3rd postoperative days, although these decreases were not significant before the operation. The authors also reported that TNF-α and IL-12 showed an increase, albeit nonsignificantly, on the 2nd postoperative day. The findings regarding the preoperative values do not agree with the results obtained in the current study.

Shahramian et al.^10^ reported that the levels of ghrelin, leptin, and TNF-α were the same in their CHD patients and controls. The authors also observed no difference between their study groups in terms of the BMI. In contrast, in our study, the serum levels of TNF-α, IL-6, and IL-18 were significantly different between the patients and controls.

Shiva et al. found that their cyanotic heart patients had a lower mean of age than their acyanotic group because of early symptoms before surgery. Both types of patients with CHD had a BMI and left ventricular ejection fraction lower than the controls, with the difference not constituting statistical significance. The authors demonstrated that their cyanotic patients had a lower mean O_2_ saturation than their acyanotic and control groups. This finding is consistent with the results of the current study, where the BMI was significantly lower in the cyanotic and acyanotic patients than in the controls.

The present study focused on both patients with CHD and control subjects. The major limitation of this study was the randomized generation of RV and PA pressures with statistical methods in the control group, which was due to constraints imposed by medical ethics.

## Conclusion

To the best of our knowledge, the present study is the first of its kind to demonstrate an association between CHD and the levels of TNF-α and some interleukins in children with CHD. Our study population is comparable to those in previous studies, although our methodology is different. We demonstrated that whereas the serum levels of TNF-α, IL-6, and IL-18 were higher among patients with CHD, especially among those suffering from cyanosis, there were no statistically significant differences with respect to IL-10 and IL-12 between the case and control groups. In addition, RV pressure was higher among our cyanotic patients. The BMI as an indicator of growth retardation was lower in the acyanotic patients, followed by the cyanotic patients, which was associated with hypoxia and remarkable left-to-right shunting. 

## Notes:

***This paper should be cited as:*** Noori NM, Shahramian I, Teimouri A, Keyvani B, Mahjoubifard M. Serum Levels of Tumor Necrosis Factor-α and Interleukins in Children with Congenital Heart Disease. J Teh Univ Heart Ctr 2017;12(1):15-22.
